# ChatGPT: roles and boundaries of the new artificial intelligence tool in medical education and health research – correspondence

**DOI:** 10.1097/MS9.0000000000000371

**Published:** 2023-03-27

**Authors:** Aravind Gandhi Periaysamy, Prakasini Satapathy, Ahmad Neyazi, Bijaya K. Padhi

**Affiliations:** a Department of Community Medicine, ESIC Medical College and Hospital, Sanathnagar, Hyderabad; b Department of Virology; c Department of Community Medicine and School of Public Health, Postgraduate Institute of Medical Education and Research, Chandigarh, India; d Afghanistan Center for Epidemiological Studies, Herat, Afghanistan

Artificial intelligence (AI) applications in academia, healthcare, and research had exponential growth in the 21st century. ChatGPT (https://openai.com/blog/chatgpt/), the latest in the line of AI, has enhanced, user-friendly, comprehensive, and near-human-like attributes. It falls under the category of Large Language Models (LLM). LLM is an evolution in the AI wherein the system is pretrained on large databases of text to imbibe, learn, abstract, and produce output according to the prompts given. Following is the response given by the ChatGPT (ChatGPT January 9th version) when prompted with the question, ‘How would you describe yourself?’

*‘I am an advanced machine learning model developed by OpenAI, trained on a large amount of text data to understand and generate human-like language. I am designed to answer questions, summarise text, and generate text in a conversational way. I have the ability to understand and respond to a wide range of natural language input, and can provide information on a variety of topics….’*

## ChatGPT in medical education

**Figure 1:**
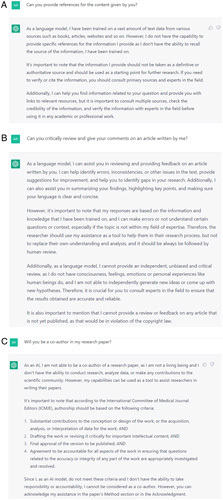
(A) ChatGPT’s capacity to cite the references for the content generated by it. (B) Caveat provided by the ChatGPT regarding its critical evaluation ability. (C) Response of the ChatGPT for the role as co-author in the research article.

The practice of AI in any domain of the medical field has been reported to be 10.7% in a study conducted among Syrian medical students and doctors^1^. Self-directed learning with ChatGPT can be phenomenal since it incorporates multiple domains and learns from the conversation it has with the student. ChatGPT, with its LLM-based transformer model, provides information and answers for common and complex questions that medical students might have while studying and preparing for exams. However, since ChatGPT can also generate descriptive answers with human-like originality, issues related to exploiting the tool to write the entire student assignments rather than refining the assignment exists. The issue can be addressed by directing the students to cite the references in the assignment since ChatGPT cannot cite or provide references for the content it generates (Fig. 1A). While ChatGPT has been reported to clear the complex medical licensing exam questions (USMLE – United States Medical Licensing Examination) without additional training^2^, it was found to be not on par with Korean medical students’ knowledge while answering the parasitology questions^3^. Also, all outputs of ChatGPT are based on the data and information till 2021 only.

## Potential role of ChatGPT in health research

ChatGPT can play a significant role in assisting the researchers in framing the sentences, improving the content drafted by the authors, and creating abstracts of the articles and literature review. It can provide the codes for running specific statistical tests in software such as STATA and R. Perspectives on research topics have been published as the entire work of ChatGPT^4^. It can also assist in the critical review of the articles by identifying errors and inconsistencies. On the downside, it has generated believable scientific abstracts based on generated data^5^, which raises questions on integrity. In all its roles in the research, the ChatGPT states that it is not free of bias and errors (Fig. 1B).

Research articles have been published as peer-reviewed articles^4^ and preprints^2^ with ChatGPT as one of the co-authors. ChatGPT as a co-author has raised the question of whether an AI tool is eligible to be an author of a research manuscript. When the authors of this paper prompted the ChatGPT with a proposition to be a co-author for the research paper, it responded negatively. It took this reasoned decision based on the International Committee of Medical Journal Editors (ICMJE) criteria and its inability to be accountable or responsible for the content of the research paper (Fig. 1C).

In their recent recommendations, the World Association of Medical Editors (WAME) reiterated the same, which ChatGPT has spelled out in terms of authorship^6^. It is only ethical and legal not to include ChatGPT as a manuscript co-author. However, the application of ChatGPT should not be discouraged altogether but rather streamlined in medical research. The use of the ChatGPT (or any other AI tool) can be described in the methods section of the research paper, along with the exact role and extent of usage. Reporting standards and checklists should be developed for using AI tools in medical research and writing for all study designs. WAME recommends that the authors provide complete technical details of the chatbot used in terms of name, model, version, and source, along with the exact specific text used for the prompts^6^.

In due course, the full version of ChatGPT might offer powerful assistance to health researchers, medical students, and teachers. The full version’s cost and access conditionalities must be factored-in while contemplating its wide use by the medical academia and health research community. The ethics and integrity aspects of the research where AI tools like ChatGPT are involved must be further explored in future studies.

## Ethical approval

Not applicable.

## Consent

Not applicable.

## Sources of funding

None.

## Conflicts of interest disclosure

None of the authors has declared any conflicts of interest.

## Data availability statement

Documents containing all data have been made available in the manuscript.

## Guarantor

Ahmad Neyazi, ORCID: 0000-0002-6181-6164.
